# Supplementary figures and images for: Alpha-Synuclein Interaction with UBL3 Is Upregulated by Microsomal Glutathione S-Transferase 3, Leading to Increased Extracellular Transport of the Alpha-Synuclein under Oxidative Stress
Source: Int J Mol Sci. 2024 Jul 4;25(13):7353. doi: 10.3390/ijms25137353 (PMC11242132; doi:10.3390/ijms25137353)

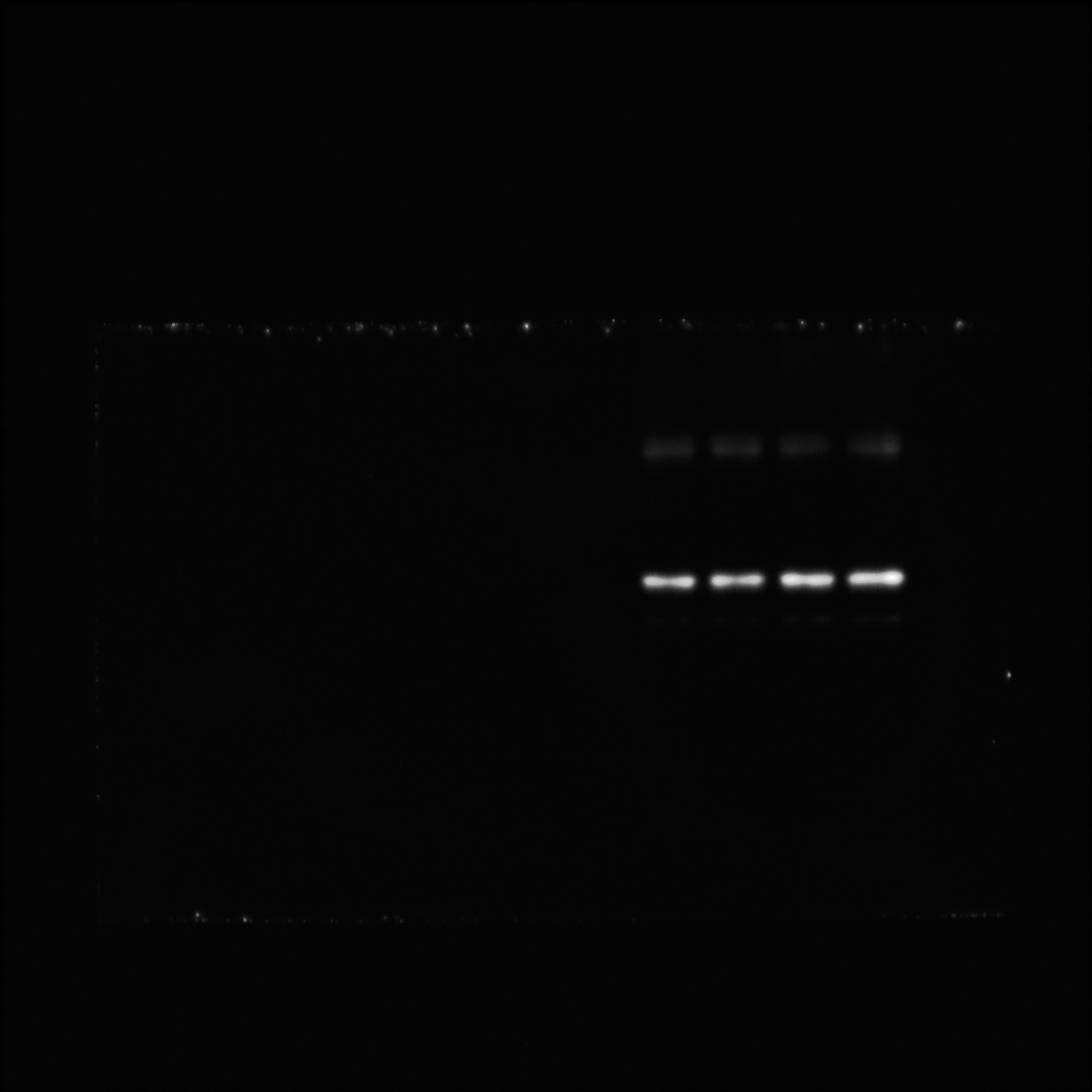

Supplement: Supplementary file 1 [file ijms-25-07353-s001.zip › a-Syn.TIF]

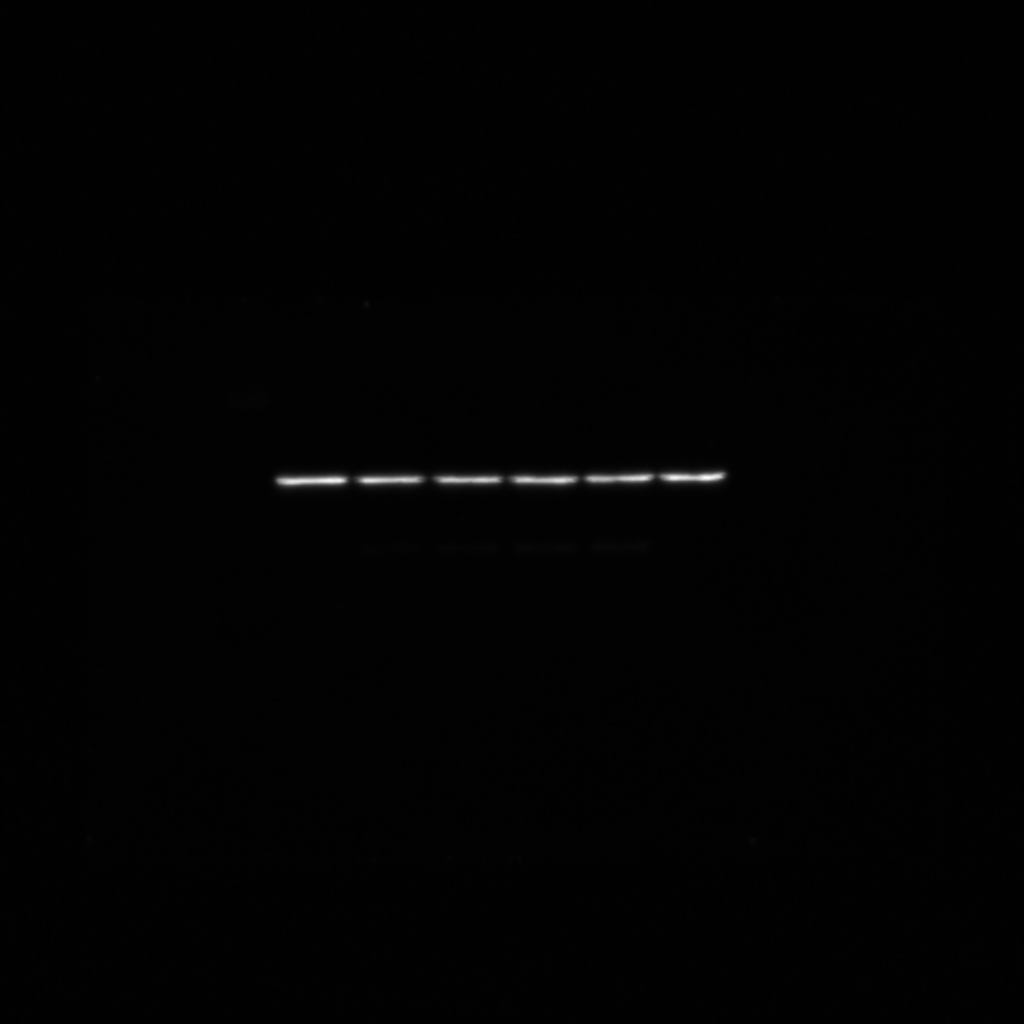

Supplement: Supplementary file 1 [file ijms-25-07353-s001.zip › B-actin.TIF]

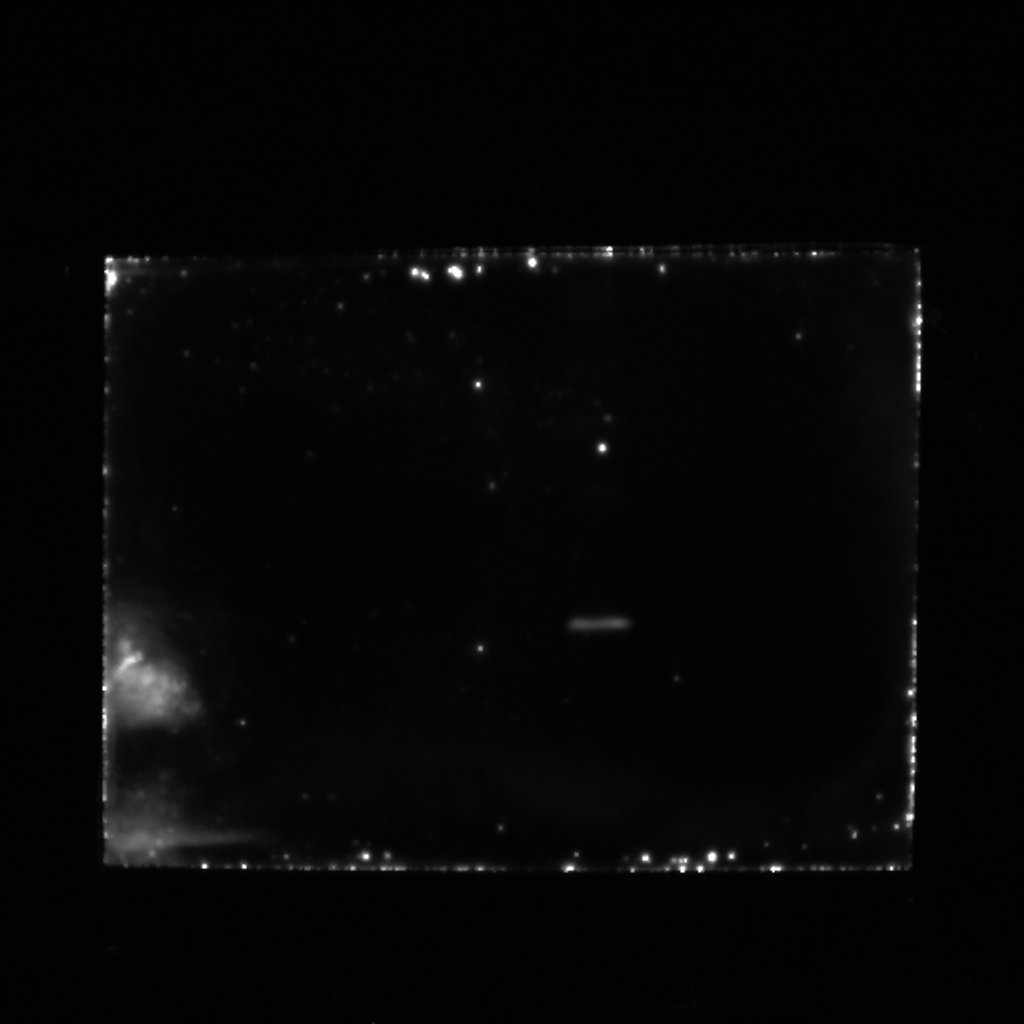

Supplement: Supplementary file 1 [file ijms-25-07353-s001.zip › HA tag.Tif]

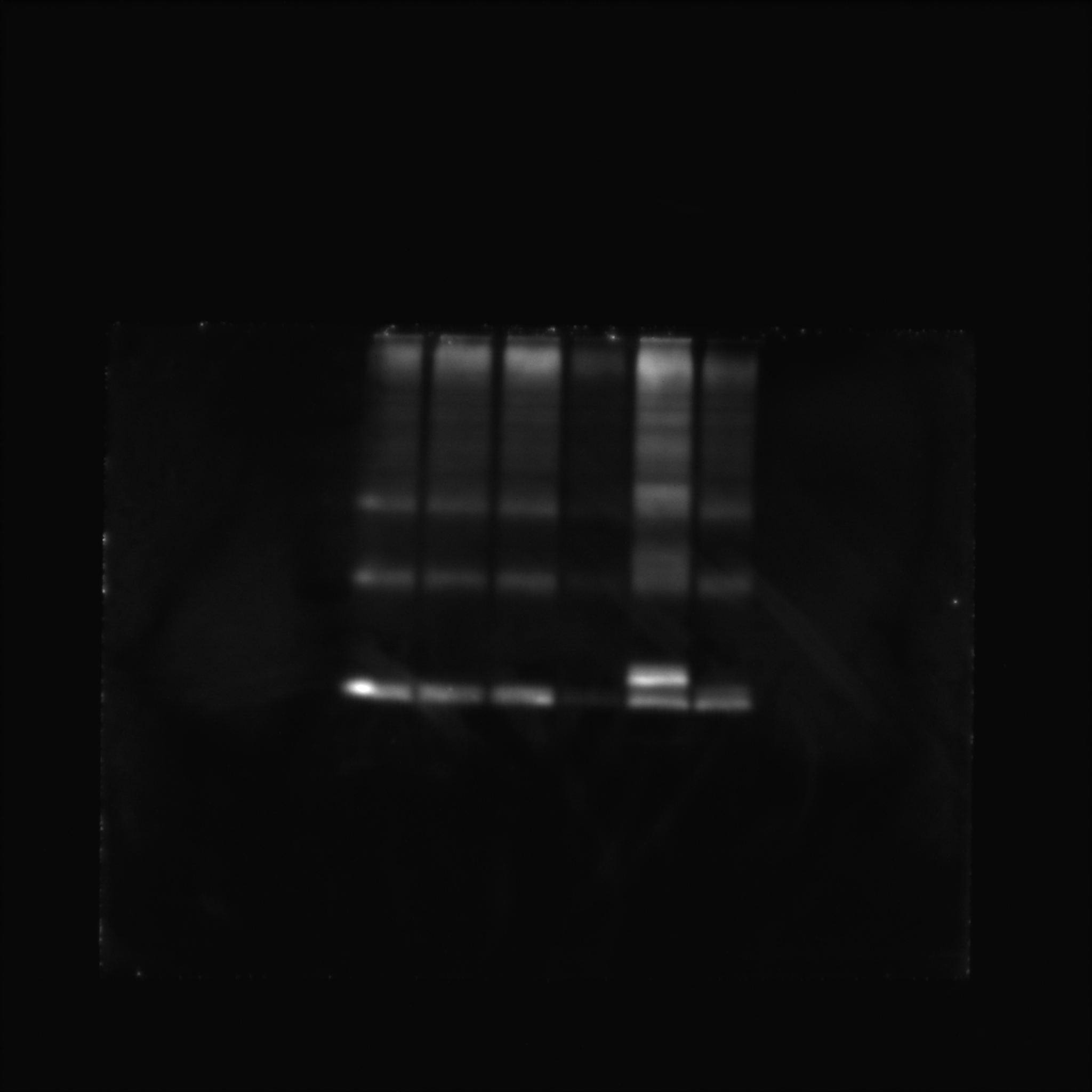

Supplement: Supplementary file 1 [file ijms-25-07353-s001.zip › MGST3.TIF]

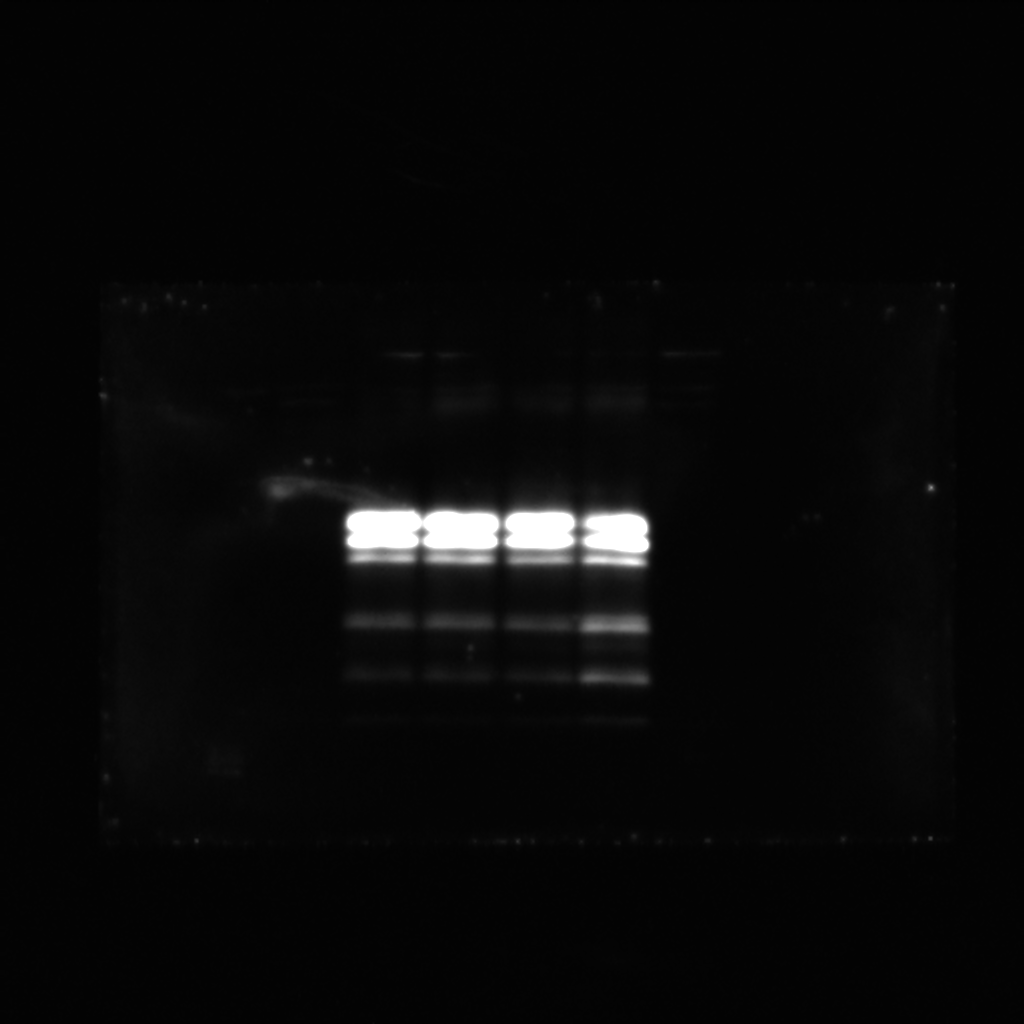

Supplement: Supplementary file 1 [file ijms-25-07353-s001.zip › UBL3.TIF]
